# Bioenergetic study of murine hepatic tissue treated *in vitro* with atorvastatin

**DOI:** 10.1186/2050-6511-14-15

**Published:** 2013-02-28

**Authors:** Ali S Alfazari, Bayan Al-Dabbagh, Saeeda Almarzooqi, Alia Albawardi, Abdul-Kader Souid

**Affiliations:** 1 Department of Internal Medicine, United Arab Emirates University, Al Ain, Abu Dhabi, United Arab Emirates; 2 Department of Pathology, United Arab Emirates University, Al Ain, Abu Dhabi, United Arab Emirates; 3 Department of Pediatrics, United Arab Emirates University, Al Ain, Abu Dhabi, United Arab Emirates

**Keywords:** Statins, Mitochondria, Respiration, Caspases, Apoptosis

## Abstract

Atorvastatin (a 3-hydroxy-3-methylglutaryl coenzyme-A reductase inhibitor) is a widely used cholesterol-lowering drug, which is recognized for its potential hepatotoxicity. This study investigated *in vitro* effects of this agent on hepatic tissue respiration, ATP content, caspase activity, urea synthesis and histology. Liver fragments from Taylor Outbred and C57Bl/6 mice were incubated at 37°C in Krebs-Henseleit buffer continuously gassed with 95% O_2_: 5% CO_2_ in the presence and absence of atorvastatin. Phosphorescence O_2_ analyzer that measured dissolved [O_2_] as a function of time was used to monitor cellular mitochondrial O_2_ consumption. The caspase-3 substrate *N*-acetyl-asp-glu-val-asp-7-amino-4-methylcoumarin was used to monitor caspase activity. The rates of hepatocyte respiration (μM O_2_ min^-1^ mg^-1^) in untreated samples were 0.15 ± 0.07 (n = 31). The corresponding rates for samples treated with 50 nM (therapeutic concentration), 150 nM or 1.0 μM atorvastatin for ≤13 h were 0.13 ± 0.05 (n = 19), *p* = 0.521. The contents of hepatocyte ATP (pmol^-1^ mg^-1^) in untreated samples were 40.3 ± 14.0 and in samples treated with 1.0 μM atorvastatin for ≤4.5 h were 48.7 ± 23.9 (*p* = 0.7754). The concentrations of urea (mg/dL mg^-1^, produced over 50 min) for untreated samples were 0.061 ± 0.020 (n = 6) and for samples treated with 1.0 μM atorvastatin for ≤6 h were 0.072 ± 0.022 (n = 6), *p* = 0.3866. Steadily, hepatocyte caspase activity and histology were unaffected by treatments with up to 1.0 μM atorvastatin for ≤6 h. Thus, the studied murine model showed preserved hepatocyte function and structure in the presence of high concentrations of atorvastatin.

## Background

Statins, 3-hydroxy-3-methylglutaryl coenzyme-A reductase inhibitors, are the most effective class of drugs that treat hypercholesterolemia. These agents reduce hepatocyte cholesterol, which results in up-regulation of the low-density lipoprotein (LDL) receptors and increased clearance of LDL-cholesterol from the plasma. Atorvastatin, (3*R*,5*R*)-7-[2-(4-fluorophenyl)-3-phenyl-4-(phenylcarbamoyl)-5-propan-2-ylpyrrol-1-yl]-3,5-dihydroxyheptanoic acid, is considered “best-in-class” for meeting the recommended treatment guidelines [1].

Elevations of liver alanine and aspartate aminotransferases (ALT and AST) are well-recognized adverse events of atorvastatin [2-4], occurring in about 0.5% of patients, usually in the first few months of therapy [5,6]. Since other lipid-lowering compounds also increase liver aminotransferases, it has been debated whether statin-associated elevated transaminases are due to hepatotoxicity or a reaction to reduced cholesterol [7]. More recent studies have shown statins are well tolerated by patients with primary biliary cirrhosis, hepatitis C and non-alcoholic steatohepatitis [8-10]. Furthermore, a few short-term studies showed statins improved hepatic inflammation in patients with non-alcoholic fatty liver disease [11]. In a prospective, double blind trial of 326 patients with chronic liver disease, fewer patients in the pravastatin group had elevations in ALT compared to placebo (7.5% *vs.* 12.5%, *p* = 0.13) [12]. The recent *post hoc* analysis of safety of atorvastatin in the Greek Atorvastatin and Coronary Heart Disease Evaluation (GREACE) study showed atorvastatin significantly ameliorated elevations of AST and ALT [13]. Other reports in humans and animals showed hepatotoxicities of statins [14], including caspase activation (apoptosis) and induction of mitochondrial disturbances [15]. These toxicities could stem from confounding factors, such idiosyncratic reactions, hypercholesterolemia, concomitant medications and co-morbidities (e.g., diabetes mellitus).

The term “cellular bioenergetics” implies the biochemical processes involved in energy metabolism (energy conversion or transformation), and the term “cellular respiration” (mitochondrial O_2_ consumption) describes the delivery of metabolites and O_2_ to the mitochondria, the oxidation of reduced metabolic fuels with passage of electrons to O_2_, and the synthesis of ATP. Impaired bioenergetics or respiration entails interferences with any of these processes.

Apoptosis describes the highly regulated mechanisms responsible for cellular responses to injuries and adverse biological signals. This process produces deleterious morphological and biochemical changes, including mitochondrial disturbances that may lead to cell death [16]. Caspases, cysteine aspartate-directed proteases and members of the interleukin-1β-converting enzyme (ICE), are key executers of apoptosis. Intracellular caspase activity can be monitored using the caspase-3 synthetic substrate *N*-cetyl-asp-glu-val-asp-7-amino-(4-methyl- coumaryl-7-amide) (Ac-DEVD-AMC). Caspase-3 is involved in proteolysis of proteins, including poly(ADP ribose) polymerase. The enzyme cleaves at the C-terminal to Asp^216^ in the asp-glu-val-asp sequence. This 4-amino-acid motif has been utilized for the highly specific caspase-3 substrate Ac-DEVD-AMC. Caspase-3 cleaves the tetrapeptide between D and AMC, releasing the fluorogenic moiety 7-amino-4-methylcoumarin (AMC). The latter can be separated on HPLC and detected by fluorescence with a great accuracy [17].

The primary aim of the study here was to investigate the effects of atorvastatin on hepatocyte bioenergetics and caspase activity. The described *in vitro* murine system allowed accurate assessment of multiple hepatic biomarkers as a function of time using murine liver tissue. The results show liver tissue function and structure are well preserved in the presence of high concentrations of atorvastatin.

## Materials and methods

### Reagents

Atorvastatin was purchased from Selleck Chemicals (Houston, TX, USA). Pd(II) complex of *meso*-tetra-(4-sulfonatophenyl)-tetrabenzoporphyrin (Pd phosphor) was purchased from Porphyrin Products (Logan, UT). A lyophilized powder of caspase inhibitor I [*N*-benzyloxycarbonyl-val-ala-asp(O-methyl)-fluoromethylketone, zVAD-fmk, *m.w.* = 467.5, a pan-caspase inhibitor] was purchased from Calbiochem (La Jolla, CA). Ac-DEVD-AMC (*N*-acetyl-asp-glu-val-asp-7- amino-4-methylcoumarin; *m.w.* = 675.64; caspase-3 substrate) was purchased from Axxora LLC (San Diego, CA). Complete® protease inhibitor cocktail was purchased from Roche Applied Science (Indianapolis, IN). Glucose (anhydrous), endotoxin- and fatty acid-free bovine serum albumin and remaining reagents were bought from Sigma-Aldrich (St. Louis, MO).

The pan-caspase inhibitor (zVAD-fmk) solution (2.14 mM) was made by dissolving 1.0 mg in 1.0 mL dimethyl sulfoxide and stored at -20°C. Ac-DEVD-AMC solution (7.4 mM) was made by dissolving 5.0 mg in 1.0 mL dimethyl sulfoxide and stored at -20°C. Pd phosphor solution (2.5 mg/mL = 2 mM) was prepared in dH_2_O and stored in aliquots at -20°C. NaCN solution (1.0 M) was prepared in dH_2_O; the pH was adjusted to ~7.0 with 12 N HCl and stored at -20°C. Glucose oxidase, 10 mg/mL in dH_2_O, was stored at -20°C. One tablet of Complete® protease inhibitor cocktail was dissolved in 1.0 mL Water-For-Injection and stored at -20°C.

### Mice

Male Taylor Outbred (TO, age: 9-10 weeks, weight: 30-35 g) and C57Bl/6 (age: 9-10 weeks, weight: 20-22 g) mice were maintained at an animal facility that was in compliance with NIH guidelines (http://grants.nih.gov/grants/olaw/references/phspol.htm). The mice were purchased from the Jackson Laboratory (Bar Harbor, ME). All mice were housed in rooms maintained at 22°C with ~60% relative humidity and a 12-hr light/dark cycle. All mice had *ad libitum* access to standard rodent chow and filtered water. All protocols here received approval from the Animal Ethics Committee-United Arab Emirates University-College of Medicine and Health Sciences.

### Liver tissue

Mice were anesthetized by sevoflurane inhalation (100 μL per 10 g body weight). Liver specimens (~20 to 30 mg) were collected using a 4-mm human skin biopsy punch (Miltex GmbH, Germany) and *immediately* immersed in 50 mL ice-cold *modified* Krebs-Henseleit (KH) buffer (115 mM NaCl, 25 mM NaHCO_3_, 1.23 mM NaH_2_PO_4_, 1.2 mM Na_2_SO_4_, 5.9 mM KCl, 1.0 mM EDTA, 1.18 mM MgCl_2_, 10 mM glucose, and 0.5 μL/mL Complete® protease inhibitor cocktail, pH 7.5) continuously gassed with 95% O_2_: 5% CO_2_[18].

The samples were then incubated *in vitro* at 37°C in 50 mL in KH buffer (115 mM NaCl, 25 mM NaHCO_3_, 1.23 mM NaH_2_PO_4_, 1.2 mM Na_2_SO_4_, 5.9 mM KCl, 1.25 mM CaCl_2_, 1.18 mM MgCl_2_, and 10 mM glucose, pH 7.5) supplemented with 0.5 μL/mL Complete® protease inhibitor cocktail and continuously gassed with 95% O_2_: 5% CO_2_. For the O_2_ measurement, specimens were placed in 1.0 mL KH buffer (air-saturated) containing 0.5% fatty acids-free bovine albumin and 3 μM Pd phosphor. Specimens were also processed for measuring caspase activity, urea synthesis and histology as described below.

### Oxygen measurement

Phosphorescence oxygen analyzer was used to monitor O_2_ consumption by liver specimens [18,19]. O_2_ detection was performed with the aid of Pd phosphor that had absorption maximum at 625 nm and phosphorescence maximum at 800 nm. Samples were exposed to light flashes (600 per min) from a pulsed light-emitting diode array with peak output at 625 nm (OTL630A-5-10-66-E, Opto Technology, Inc., Wheeling, IL). Emitted phosphorescent light was detected by a Hamamatsu photomultiplier tube (928) after first passing it through a wide-band interference filter centered at 800 nm. The amplified phosphorescence decay was digitized at 1.0 MHz by a 20-MHz A/D converter (Computer Boards, Inc., Mansfield, MA).

A program was developed using Microsoft Visual Basic 6, Microsoft Access Database 2007, and Universal Library components (Universal Library for Measurements Computing Devices; http://www.mccdaq.com/daq-software/universal-library.aspx). It allowed direct reading from the PCI-DAS 4020/12 I/O Board (PCI-DAS 4020/12 I/O Board; http://www.mccdaq.com/pci-data-acquisition/PCI-DAS4020-12.aspx). The pulse detection was accomplished by searching for 10 phosphorescence intensities >1.0 volt (by default). Peak detection was accomplished by searching for the highest 10 data points of a pulse and choosing the data point closest to the pulse decay curve [20].

The phosphorescence decay rate (1/τ) was characterized by a single exponential; I = Ae^-*t*/τ^, where I = Pd phosphor phosphorescence intensity. The values of 1/τ were linear with dissolved O_2_: 1/τ = 1/τ^o^ + *k*_*q*_[O_2_], where 1/τ = the phosphorescence decay rate in the presence of O_2_, 1/τ^o^ = the phosphorescence decay rate in the absence of O_2_, and *k*_q_ = the second-order O_2_ quenching rate constant in s^-1^ • μM^-1^.

Respiration was measured at 37°C in 1-mL sealed vials. Mixing was with the aid of parylene-coated stirring bars. In vials sealed from air, [O_2_] decreased linearly with time, indicating the kinetics of mitochondrial O_2_ consumption was zero-order. The rate of respiration (*k*, in μM O_2_ min^-1^) was thus the negative of the slope d[O_2_]/d*t*. NaCN inhibited respiration, confirming O_2_ was consumed in the mitochondrial respiratory chain.

The calibration reaction contained PBS with 3 μM Pd phosphor, 0.5% fat-free albumin, 50 μg/mL glucose oxidase and various concentrations of β-glucose. [O_2_] was calculated using, 1/τ = 1/τ^o^ + *k*_*q*_[O_2_] [21]. Rates of cellular respiration were normalized per mg of liver tissue (i.e., expressed as μM O_2_ consumed per min per mg liver tissue).

### ATP content

Liver fragments were homogenized in ice-cold 2% trichloroacetic acid for 2 min and neutralized with 100 mM Tris-acetate, 2 mM EDTA, pH 7.75. The supernatant was collected by centrifugation (1000 × *g* at 4°C for 5 min) and stored at -20°C until analysis. The pH of samples was adjusted to 7.75 immediately before ATP determination. ATP concentration was measured using the Enliten ATP Assay System (Bioluminescence Detection Kit, Promega, Madison, WI). Briefly, 2.5 μL of the acid-soluble supernatant was added to 25 μL of the luciferin/luciferase reagent. The luminescence intensity was measured at 25°C using Glomax Luminometer (Promega, Madison, WI). The ATP standard curve was linear from 10 pM to 100 nM (*R*^*2*^ >0.9999).

### Intracellular caspase activity

Liver specimens were incubated at 37°C in oxygenated KH buffer containing 37 μM Ac-DEVD-AMC with and without 32 μM zVAD-fmk (final volume, 0.5 mL). The tissue was then disrupted by vigorous homogenization and 10 passages through a 27-G needle. The Ac-DEVD-AMC cleavage reaction was quenched with tissue disruption, mainly because caspases became inactive due to dilution. The supernatant was collected by centrifugation (16,300 *g* for 90 min) through a Microcentrifuge Filter (nominal molecular weight limit = 10,000 Dalton, Sigma^©^), separated on HPLC, and analyzed for the free fluorogenic AMC moiety. The elution time for AMC was about 5.0 min.

### HPLC

The analysis was performed on a Waters 1525 reversed-phase HPLC system (Spectra Lab Scientific Inc, Alexandria, VA) that consisted of manual injector, pump and fluorescence detector. The excitation wavelength used was 380 nm and the emission wavelength 460 nm. Solvents A and B were HPLC-grade CH_3_OH: dH_2_O (1:1; isocratic). The Ultrasphere IP column (4.6 × 250 mm, Beckman) was operated at 25°C at 1.0 mL/min. The run time was 20 min. The injection volume was 50 μL.

### Urea synthesis

Liver specimens were incubated at 37°C in 50 mL KH buffer (continuously gassed with 95% O_2_: 5% CO_2_) with and without 1.0 μM atorvastatin for up to 6 h. Specimens were then removed from the incubation solution every 60 min and placed in 1.0 mL KH buffer supplemented with 10 mM NH_4_Cl and 2.5 mM ornithine. The reactions were allowed to continue at 37°C for 50 min with continuous gassing as above. At the end of the incubation period, the solutions were analyzed for urea as described [22]. Blood urea nitrogen (BUN, mmol/L) was measured using standard laboratory methods with an LX20 multiple automated analyzer (Beckman Coulter, CA, USA). For conversion, BUN (mg/dL) = BUN (mmol/L) ÷ 0.357; Urea (mg/dL) = BUN (mg/dL) × 2.14.

### Histology

Liver samples were fixed in 10% neutral formalin, dehydrated in increasing concentrations of ethanol, cleared with xylene and embedded in paraffin. Four-micrometer sections were prepared from paraffin blocks and stained with hematoxylin and eosin.

### Statistical analysis

Data were analyzed using SPSS statistical package (version 19). The nonparametric test (2 independent variables) Mann-Whitney was used to compare treated and untreated samples. Respiration rates (*k*_*c*_, in μM O_2_ min^-1^ mg^-1^), cellular ATP content (pmol mg^-1^), AMC peak areas (arbitrary unit mg^-1^) and urea (mg/dL per mg liver tissue) for untreated samples were compared with those for treated samples.

## Results

### Bioenergetics of liver tissue treated with atorvastatin

**Figure 1:**
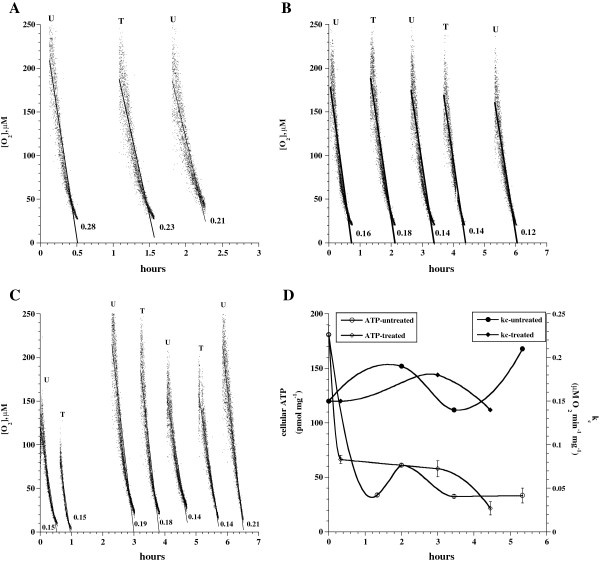
**Atorvastatin neither alters hepatocyte respiration nor hepatocyte ATP content.** Panels **A-C**: Cellular mitochondrial O_2_ consumption with and without atorvastatin. Panels **A-B**, O_2_ runs with and without 150 nM atorvastatin. Panel **C**, O_2_ runs with and without 1.0 μM atorvastatin. Panel **D**: Cellular ATP content and values of *k*_*c *_ as a function of incubation time. Liver specimens from TO mice were incubated *in vitro* at 37°C in 50 mL KH buffer (continuously gassed with 95% O_2_: 5% CO_2_) with and without 150 nM (**A-B**) or 1.0 μM (**C-D**) atorvastatin. Cellular O_2_ consumption and ATP content were determined as a function of time; *t* = 0 corresponded to tissue collection. The lines in Panels **A-C** are linear fits (0.873 < *R*^2^ < 0.955). The rate of respiration (*k,* μM O_2_ min^-1^) was set as the negative of the slope of [O_2_] *vs. t*. The values of *k*_*c *_ (μM O_2_ min^-1^ mg^-1^) are shown at the bottom of each run. The values of *k*_*c *_ in Panel **C** and the cellular ATP content of the same experiment are plotted in Panel **D**. Eleven independent experiments were done with the TO mice and 4 independent experiments were done with the C57Bl/6 mice. U, untreated; T, treated.

To assess the effects of atorvastatin on liver tissue bioenergetics, specimens from ten Taylor Outbred (TO) mice and three C57Bl/6 mice were incubated at 37°C with 50 nM (therapeutic concentration; Maier et al. [23]), 150 nM or 1.0 μM atorvastatin and analyzed for cellular O_2_ consumption and ATP content as a function of time. Results of representative experiments are shown in Figure 1.

In Figure 1A-B, liver specimens from TO mice were incubated at 37°C in 50 mL KH buffer (continuously gassed with 95% O_2_: 5% CO_2_) with and without 150 nM atorvastatin for up to 6 h. Samples were alternatively removed from the incubation mixture and processed for O_2_ measurement at 37°C. The rate of respiration (*k,* μM O_2_ min^-1^) was set as the negative of the slope of [O_2_] *vs. t*. The values of *k*_*c*_ (μM O_2_ min^-1^ mg^-1^; mean ± SD) for untreated samples were 0.18 ± 0.06 (n = 5, for *t* from 1.8 to 5.3 h) and for treated samples 0.18 ± 0.05 (n = 3, for *t* from 1.1 to 3.7 h), *p* = 0.9737.

Liver samples were also incubated as above with and without 50 nM atorvastatin. The value of *k*_*c*_ for untreated tissue was 0.15 μM O_2_ min^-1^ mg^-1^ (*t* = 1.4 h) and for treated tissue 0.15 μM O_2_ min^-1^ mg^-1^ (*t* = 2.2 h). In 10 independent experiments involving incubations with 50 nM, 150 nM or 1.0 μM atorvastatin for up to 13 h, the rates of respiration for untreated specimens were 0.15 ± 0.07 (n = 31 runs) and for treated specimens 0.13 ± 0.05 (n = 19 runs), *p* = 0.521.

In C57Bl/6 strain, the value of *k*_*c*_ for untreated tissue was 0.10 ± 0.03 (1.8 < *t* < 5.3 h, n = 5) and for tissue treated with 1.0 μM atorvastatin 0.11 ± 0.04 (for *t* from 1.1 to 3.7 h, n = 4). In another experiment, the value of *k*_*c*_ for untreated tissue was 0.12 ± 0.05 (for *t* from 1.3 to 5.9 h, n = 4) and treated tissue 0.12 ± 0.04 (for *t* from 0.5 to 4.4 h, n = 3). Thus, hepatocyte respiration was preserved in the presence of high doses of atorvastatin for up to 13 h.

In Figure 1C-D, liver samples were incubated as above with and without 1.0 μM atorvastatin for up to 6.5 h and processed for measurements of cellular respiration and ATP content. The results are plotted as a function of incubation time in Panel D. The values of *k*_*c*_ for untreated samples (for *t* from 0 to 5.3 h) were 0.17 ± 0.03 μM O_2_ min^-1^ mg^-1^ and for treated samples (for *t* from 0 to 4.5 h) 0.16 ± 0.02 μM O_2_ min^-1^ mg^-1^ (*p* = 0.3954). Cellular ATP at *t* = 0 h was 181.1 ± 8.0 pmol mg^-1^. For untreated specimens, cellular ATP (in pmol mg^-1^, measured in triplicates) at *t* = 1.3 h was 33.9 ± 1.7, at *t* = 2.0 h was 61.2 ± 1.4, at *t* = 3.5 h was 32.6 ± 1.4, and at *t* = 5.3 h was 33.4 ± 6.8. For treated specimens, cellular ATP at *t* = 0.3 h was 66.5 ± 3.9, at *t* = 3.0 h was 58.0 ± 7.4 and at *t* = 4.5 h was 21.6 ± 6.1. Thus, the overall ATP contents for untreated samples (for *t* from 1.3 to 5.3 h) were 40.3 ± 14.0 and for treated samples (for *t* from 0.3 to 4.5 h) 48.7 ± 23.9 (*p* = 0.7754). In another experiment, ATP contents at 6 h for untreated tissue were 10.5 ± 1.0 (n = 4) and for treated tissue 16.6 ± 0.9 (n = 4). Thus, hepatocyte ATP was highest immediately after tissue collection (*in vivo* levels of hepatocyte bioenergetics) and declined equally in the presence and absence of atorvastatin (*in vitro* levels of hepatocyte bioenergetics).

### Hepatocyte caspases in liver tissue treated with atorvastatin

**Figure 2:**
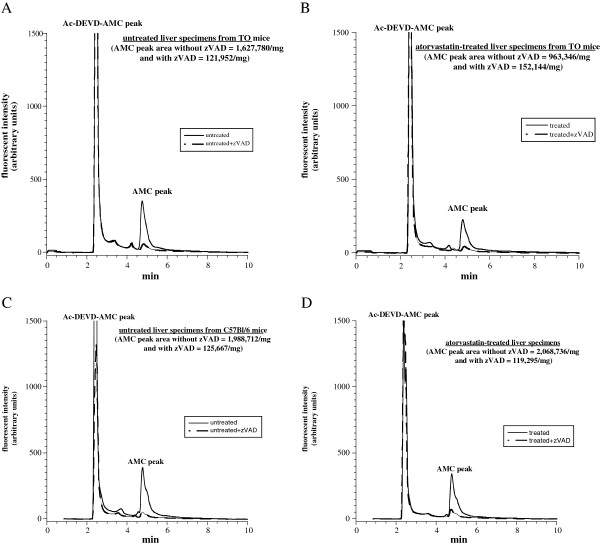
**Atorvastatin does not induce hepatocyte caspases.** Panel **A**, untreated liver specimens from TO mice. Panel **B**, atorvastatin-treated liver specimens from TO mice. Panel **C**, untreated liver specimens from C57Bl/6 mice. Panel **D**, atorvastatin-treated liver specimens from C57Bl/6 mice. Representative experiments of liver specimens incubated *in vitro* at 37°C with (B and D) and without (A and C) 1.0 μM atorvastatin for 6 hr are shown. At the end of incubation period, the samples (24.7 to 35.1 mg) were rinsed and incubated at 37°C in 1.0 mL oxygenated KH buffer with and without 32 *μ*M zVAD-fmk for 10 min. Ac-DEVD-AMC (37 *μ*M) was then added and the incubation continued for 20 min. The tissues were vigorously disrupted and the supernatants were separated on HPLC and analyzed for the AMC (*R*_*t*_, ~5.0 min) and Ac-DEVD-AMC peaks (*R*_*t*_, ~2.5 min). Eleven independent experiments were done with the TO mice and 4 independent experiments were done with the C57Bl/6 mice.

Representative experiments for hepatocyte caspase activity in TO (Panels A-B) and C57BL/6 (Panels C-D) strains are shown in Figure 2. The samples were incubated at 37°C with and without 1.0 μM atorvastatin for 6 h. The specimens were then incubated at 37°C with 37 μM Ac-DEVD-AMC in the presence and absence of zVAD-fmk (32 μM). In untreated samples from the TO strain (Panel A), the AMC peak area (arbitrary unit, reflecting caspase activity) without zVAD was 1,627,780 mg^-1^ and with zVAD was 121,952 mg^-1^ (93% inhibition). In treated samples (Panel B), the AMC peak area without zVAD was 963,346 mg^-1^ and with zVAD was 152,144 mg^-1^ (84% inhibition). In untreated samples from the C57BL/6 strain (Panel C), the AMC peak area without zVAD was 1,988,712 mg^-1^ and with zVAD 125,667 mg^-1^ (94% inhibition). In treated samples (Panel D), the AMC peak area without zVAD was 2,068,736 mg^-1^ and with zVAD 119,295 mg^-1^ (94% inhibition). Thus, hepatocyte caspase activity at 6 h was similar in untreated and treated samples.

### Urea synthesis by liver tissue treated with atorvastatin

Liver specimens were incubated at 37°C in 50 ml KH buffer (continuously gassed as above) with and without 1.0 μM atorvastatin for 6 hr. Every 60 min, specimens (sum sample weight = 86.9 ± 5.9 mg) were removed from the incubation solutions and placed in 1.0 mL KH buffer supplemented with 10 mM NH_4_Cl and 2.5 mM ornithine. The solutions were then analyzed for urea at min 50. The concentrations of urea (mg/dL per mg liver tissue) for untreated and treated samples were not significantly different (Table 1).

**Table 1:** **Urea synthesis (mg/dL mg**^**-1**^**, produced over 50 min) by liver tissue treated with atorvastatin**

**Untreated (n =6)**	**Treated (n =6)**	***P*-value**
0.061 ± 0.020	0.072 ± 0.022	0.3866

Liver specimens were incubated at 37°C in KH buffer with and without 1.0 μM atorvastatin for 6 hr. Every 60 min, specimens were removed from the incubation solutions and placed in 1.0 mL KH buffer supplemented with 10 mM NH_4_Cl and 2.5 mM ornithine. The solutions were then analyzed for urea at min 50. The values are mean ± SD for hr 1 to 6.

### Histology

**Figure 3:**
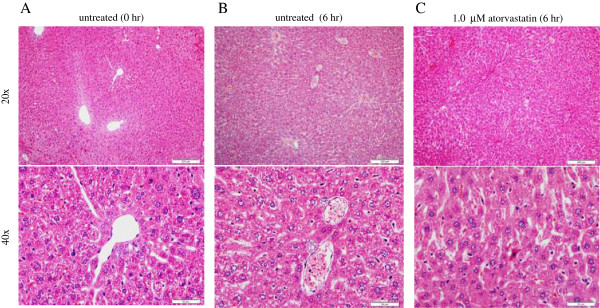
**Micrographs of hematoxylin and eosin-stained liver sections from untreated and atorvastatin-treated TO mouse.** Results of representative experiment of liver specimens incubated *in vitro* at 37°C with and without 1.0 μM atorvastatin for 6 hr is shown. A liver specimen at 0 hr is also shown. Liver structure and cytology are preserved in treated and untreated specimens. Inflammation and cholestasis are absent. (Hematoxylin and eosin, 10× and 40×).

Representative micrographs of hematoxylin and eosin stained sections of untreated tissue at 0 and 6 hr and tissue treated with 1.0 μM atorvastatin at 6 hr are shown in Figure 3. The incubation conditions were as above. Liver architecture and cytology were preserved in treated and untreated specimens. Inflammation and cholestasis were absent.

## Discussion

Asymptomatic increase in hepatic transaminases is most common adverse event of atorvastatin, occurring in about 0.5% of patients [1]. Other hepatocellular injuries, such as cholestasis, immune hepatitis and fulminant liver failure are also possibly linked to atorvastatin use (Bhardwaj and Chalasani [7]; see also atorvastatin product insert). The duration between exposure and onset of toxicity varies, ranging from 12 hours to 52 weeks. The transaminase elevations, however, are frequently dose-dependent and occur in the first 16 weeks of therapy [3,24].

Oxygen consumption is sensitive to reduced cellular metabolic fuels, as well as to mitochondrial derangements. Cellular respiration is reduced in the presence of nutrient depletion or electron transport chain deficits. The rate of respiration, on the other hand, is enhanced in the presence of proton leak (uncoupling oxidative phosphorylation). As shown previously, measurements of cellular mitochondrial oxygen consumption by the phosphorescence oxygen analyzer are highly sensitive to these cellular insults [17]. Moreover, liver architecture and urea synthesis are typical biomarkers for assessing hepatocyte injury [25].

Hepatotoxicities were evident in diabetic and hypercholesterolemic rats treated with oral atorvastatin (5 mg/kg daily) for two months [14]. Several reports also described statin-induced mitochondrial toxicities (see Dykens and Will [26]). More recently, impaired mitochondrial oxidative phosphorylation, membrane fluidity and coenzyme Q (ubiquinone, a component of the mitochondrial respiratory chain) content were reported in rats treated with 80 mg/kg atorvastatin for 4 weeks. The authors suggested that impaired hepatocyte bioenergetics may play a role in the development of statin-induced hepatotoxicities [15].

Atorvastatin also exerts cytotoxic effects on human hematopoietic tumors by promoting apoptosis through the mitochondrial cell death pathway. Other potential mechanisms involve altering the membrane localization of small GTPases. Treatment with statin resulted in reduction of mitochondrial membrane potential and cytosolic release of the activator of caspases Smac/DIABLO. As a result, caspases 9, 3 and 8 were efficiently activated [27].

This study investigated whether atorvastatin impairs hepatocyte cellular bioenergetics (respiration and ATP content). Strain-specific drug toxicities have been described [28]. Therefore, our animal model always tests different murine strains or a murine strain and a rat strain. The results clearly show preserved murine hepatocyte respiration and ATP content following *in vitro* exposure to 1.0 μM atorvastatin for several hours (Figure 1). This concentration is about 20-fold higher than therapeutic plasma levels. Consistently, hepatocyte caspase activity (Figure 2) and liver architecture (Figure 3) are preserved in the presence of 1.0 μM atorvastatin and indeed inflammation and cholestasis were absent. Thus, in the studied murine hepatic model, atorvastatin was not toxic. It is unknown, however, whether a much longer exposure produces cytotoxicity and will definitely be a venture for future research.

Cellular ATP was highest *immediately* after tissue collection (reflecting *in vivo* levels of bioenergetics) and declined subsequently to a new steady state (reflecting *in vitro* levels of bioenergetics), Figure 1D. This assumption is consistent with the preserved hepatocyte structure (Figure 3) and ultrastructure (data not shown) following tissue collection. Along the same lines, about 50% decline in cortical ATP levels was noted in rat brain 15 min following cortical surgery [29]. The *in vitro* levels of ATP shown in Figure 1D, however, are much higher than those reported for cultured rat hepatocytes at the steady state level (2.44 ± 0.09 pmol mg^-1^) [25].

Urea synthesis is a sensitive biomarker for hepatocellular functions. As shown in Table 1, the presence of atorvastatin for up to 6 hr had no significant effect on the rate of hepatocyte urea synthesis. Consistently, hepatocyte structure was preserved in the presence of atorvastatin (Figure 3).

Statins inhibit the rate-limiting step of cholesterol biosynthesis catalyzed by HMG-CoA reductase. This inhibition leads to decreased hepatic cholesterol synthesis, up-regulation of low-density lipoprotein (LDL) receptor, and increased clearance of plasma LDL-cholesterol. As a result of inhibiting HMG-CoA reductase, statins could also inhibit the synthesis of important isoprenoids, such as farnesylpyrophosphate (FPP) and geranylgeranylpyrophosphate (GGPP). These intermediates serve as lipid attachments for the post-translational modification of intracellular proteins, such as nuclear lamins, Ras, Rho, Rac and Rap [30]. Hence, the pleiotropic effects of statins may arise from combined cholesterol lowering effects and inhibition of intracellular isoprenoid-dependent proteins.

## Conclusion

In conclusion, this *in vitro* study shows that murine hepatocyte bioenergetics, caspase activity and histology are preserved in the presence of high concentrations of atorvastatin for at least 6 hours. These observations are consistent with the fact that long term atorvastatin therapy is well tolerated clinically [8-10].

In pre-clinical drug development, *in vitro* studies are routinely performed prior to *in vivo* testing. Due to potential pharmacodynamic differences, *in vitro* pharmacological studies should be followed by *in vivo* testing. The biomarkers (hepatocyte bioenergetics and caspase activity) described in this study, however, can be easily adapted for *in vivo* studies. Moreover, due to potential species differences in response to atorvastatin, liver tissue from small and large animals (e.g., rats and monkeys) are needed for better prediction of organ toxicity.

## Competing interests

The authors declare that they have no competing interests.

## Authors’ contributions

ASA and BA designed the study, carried out the analysis, interpreted the data and drafted the manuscript. SA and AA performed the histology. AKS supervised the progress and critically revised the manuscript. All authors read and approved the final manuscript.

## Pre-publication history

The pre-publication history for this paper can be accessed here:

http://www.biomedcentral.com/2050-6511/14/15/prepub
